# Psychological care for cancer survivors: a 2 × 2 model of interpersonal emotion regulation by caregivers

**DOI:** 10.3389/fpsyg.2024.1390692

**Published:** 2024-06-24

**Authors:** Zihao Zeng, Karen Holtmaat, Sander L. Koole

**Affiliations:** ^1^Department of Clinical, Neuro and Developmental Psychology, Vrije Universiteit Amsterdam, Amsterdam, Netherlands; ^2^Amsterdam Public Health, Mental Health, Amsterdam, Netherlands; ^3^School of Educational Science, Hunan Normal University, Changsha, China

**Keywords:** psychological care, cancer survivors, paternalistic, assertive, instrumental, supportive, interpersonal emotion regulation

Caregivers make a vital contribution to the emotional wellbeing of cancer survivors (Fong et al., 2017; Li et al., 2018; Harms et al., 2019). A recent systematic review comprising 86 studies with over 69,000 cancer survivors revealed that various forms of interpersonal emotion regulation by caregivers, such as providing emotional support or giving a warm embrace, are positively associated with a broad spectrum of mental health indicators, including less distress, anxiety, and depression, along with a better quality of life and overall wellbeing (Zeng et al., under review).^1^ Though this is a sizable body of evidence, this finding tacitly adopts the cancer survivor's perspective as the recipient of social-emotional support. Consequently, the perspective of caregivers in regulating survivors' and their own emotions remains understudied. Similar to survivors, caregivers have to cope with fear and uncertainty about the future. They may be confronted with complicated caregiving tasks and long-lasting role changes in the relationship (LeSeure and Chongkham-Ang, 2015). In the present study, we have addressed some of the psychological complexities in interpersonal emotion regulation by caregivers of cancer survivors.

## A 2 × 2 model of interpersonal emotion regulation by caregivers to cancer survivors

Improving the wellbeing of cancer survivors is a central concern for caregivers, often achieved by fostering pleasant, or hedonic, emotions—relieving the cancer survivor's suffering and improving their good spirits. However, there are situations where promoting others' broader wellbeing may be accompanied by momentary discomfort (Niven et al., 2009). In such cases, caregivers may intentionally lead the survivor to feel worse. Zaki (2020) has characterized the latter form of interpersonal emotion regulation as paternalistic because regulators assume they know what is best for the person whose emotions they are trying to influence.

Zaki (2020) has further distinguished empathically based altruistic motives, often accompanied by feelings of love or companionship, as drivers of interpersonal emotion regulation. Although altruism is an important source of motivation in caring for cancer survivors, caregivers also have their own needs. The task of caring for a cancer survivor is emotionally taxing and often carried out over the years (Kim and Given, 2008; Kent et al., 2016; Üzar-Özçetin and Dursun, 2020). Additionally, caregivers often have to grapple with their own emotional vulnerabilities, such as sadness or existential fears. Such self-serving motivations can be legitimate but may conflict with the immediate interests of cancer survivors. The latter, paternalistic and self-centric aspects of interpersonal emotion regulation have, to date, received little attention in research on care for cancer survivors.

Similar to survivor-centric (altruistic) motivations for interpersonal emotion regulation, caregiver-centric (self-serving) motivations may target both the hedonic and counter-hedonic emotional states of cancer survivors. To serve their own emotional needs, caregivers sometimes evoke positive and sometimes negative emotions in cancer survivors. When we combine caregivers' motivations with the target emotions of cancer survivors, four caregiver orientations emerge. The resulting model of interpersonal emotion regulation by caregivers for cancer survivors is summarized in Table 1. Notably, these orientations are ideal types that are separated only for analytical purposes. In real-life situations, altruistic and self-serving motives can be expected to co-occur, and changes in situational demands may prompt caregivers to shift between hedonic and counter-hedonic regulation. In everyday life, caregivers' behavior is thus likely to be a blend of these different orientations.

**Table 1:** A 2 × 2 model of interpersonal emotion regulation by caregivers to cancer survivors.

		**Caregiver's motivation**	
		**Survivor-centric**	**Caregiver-centric**
The survivor's target emotional state	Hedonic	*Supportive*	*Instrumental*
		^*^Companionship	^*^Avoid witnessing distress
		^*^Affectionate support	^*^Protective buffering
	Counter-hedonic	*Paternalistic*	*Assertive*
		^*^Invoking war metaphors to carry on despite discomfort	^*^Empathy avoidance
		^*^Invoking anxiety to promote medical adherence	^*^Blaming and guilting behaviors

Examples of each type of interpersonal emotion regulation are marked by an asterisk (^*^).

## Applying the 2 × 2 model

The 2 × 2 model depicted in Table 1 is novel and thus still awaits systematic empirical testing. Nonetheless, in the following sections, we demonstrate the utility of the model by considering how it may also serve as an integrative framework for existing research findings.

## Survivor-centric regulation: supportive and paternalistic ideal types

Survivor-centric interpersonal emotion regulation is often aimed at making cancer survivors feel better. This form of supportive regulation has, to date, been the main focus of research on interpersonal emotion regulation among cancer survivors (Zeng et al., under review) (see text footnote ^1^). As shown in the top left quadrant of Table 1, examples include offering companionship (Thomas et al., 2002) and affectionate support, i.e., physical demonstrations of love and care (Alison Payne et al., 2008). Although these supportive strategies are important and highly meaningful, not all survivor-centric interpersonal emotion regulations are aimed at promoting more positive hedonic states in cancer survivors.

More specifically, the paternalistic type of interpersonal emotion regulation seeks to evoke more negative emotions. Caregivers do not do this because they want to make cancer survivors suffer, but rather because they believe that certain negative emotions may have instrumental benefits for cancer survivors. As depicted in the bottom left quadrant of Table 1, one negative emotion that caregivers may strive to promote in cancer survivors is anger. For instance, it is well documented that many caregivers use war metaphors to describe living and coping with cancer (Penson et al., 2004; Semino et al., 2018). To carry on, caregivers may encourage survivors to disregard inconveniences in the present and to firmly focus on fighting and getting through this period. While cancer survivors are often put off by war metaphors (Semino et al., 2018), caregivers might still want to use them because they believe that anger and aggressiveness can mobilize survivors' energies in facing challenges.

Another negative emotion that caregivers may sometimes seek to induce in cancer survivors is (mild) anxiety. Anxiety is known to promote watchfulness (Derakshan and Eysenck, 2009). Consequently, when cancer survivors are not sufficiently watchful, caregivers might attempt to instill mild levels of anxiety in cancer survivors to ensure that the latter engage in necessary preventive behaviors, such as regular check-ups and medication adherence (Oliveria et al., 2013; Seibel et al., 2023). A qualitative study among 25 German survivors after curative lung cancer treatment and 17 caregivers on cancer follow-up perceptions revealed that many caregivers encourage cancer survivors to undergo regular health checks, even when these evoke “Scanxiety” among cancer survivors (Seibel et al., 2023). Overall, though research on these topics is scarce, some initial evidence that caregivers engage in paternalistic forms of interpersonal emotion regulation are available.

## Caregiver-centric regulation: instrumental and assertive ideal types

There is a large body of research on caregiver burdens (Liu et al., 2020). Nonetheless, caregiver-centric motives for interpersonal emotion regulation have, to date, not received much attention. In general, well-adjusted relationships always involve a joint consideration of one's own and others' interests (Helgeson and Fritz, 2000; Oakley, 2013). It is in the best interest of both the survivors and caregiver, particularly in the long run, that caregivers appropriately attend to their own emotional needs (Lambert et al., 2012; Girgis et al., 2013; Sklenarova et al., 2015). Addressing caregiver-centric motivations is therefore potentially useful in maintaining high-quality care for cancer survivors.

Caregiver-centric interpersonal emotion regulation may be aimed at enhancing positive emotions in cancer survivors. For instance, caregivers may sometimes find it hard to witness cancer survivors' emotional distress and may, at least from time to time, want to avoid being confronted with it. The self-serving motivation to escape survivors' distress is psychologically distinct from the altruistic motive to alleviate another person's suffering (Batson et al., 1987). Caregivers may thus seek to provide emotional comfort to cancer survivors in order to they feel better themselves. As noted in the lower right quadrant of Table 1, one example of such instrumental regulation is protective buffering, defined as “withholding or denying cancer-related thoughts and concerns from one's partner, hiding dispiriting information, and acquiescing to avoid conflict” (Langer et al., 2009, p. 4312). Although protective buffering might superficially appear altruistic, it is often used by caregivers to protect themselves from personal negative feelings from upsetting the cancer survivor (Langer et al., 2009). Unfortunately, this instrumental form of interpersonal emotion regulation may unintentionally increase the psychological distance between the caregiver and cancer survivor (Winterheld, 2017).

Finally, the assertive type of interpersonal emotion regulation aims to induce counter-hedonic emotional states in cancer survivors to enhance the feelings of the caregiver. Because it may cause emotional discomfort among cancer survivors, the assertive type is probably the most controversial form of interpersonal emotion regulation. However, there are situations where assertive regulation is at least somewhat legitimate. Caring for cancer survivors imposes significant burdens on caregivers, especially when this responsibility extends over an extended period, which is increasingly common (Kim and Given, 2008; Guerra-Martín et al., 2023). To be able to carry these burdens, caregivers must address their own needs, even if, at least in the short run, this causes emotional discomfort for cancer survivors. Two illustrative examples of the assertive type are shown on the lower right side of Table 1.

One form of assertive interpersonal emotion regulation may be empathy fatigue, a phenomenon in which caregivers experience a gradual decline in empathy toward cancer survivors (see also Cavanagh et al., 2020; Shi et al., 2022). A study of 117 cancer healthcare professionals in Ireland indicates that over a quarter of cancer care professionals report a certain level of empathy fatigue (Hunt et al., 2019). Empathy fatigue may be a protective mechanism that prevents emotional exhaustion in caregivers (Lelorain et al., 2012; see also Tops et al., 2015). Another instance of assertive interpersonal emotion regulation may occur when caregivers engage in guilting and blaming behaviors toward cancer survivors. A study involving 304 Canadian dyads of lung cancer survivors and caregivers observed that caregivers were more inclined to blame survivors, especially if they continued to smoke (Lobchuk et al., 2012). Such blaming tendencies may negatively impact the quality of caregiving but may still serve an adaptive role, perhaps by allowing caregivers and cancer survivors to achieve a more balanced give-and-take in their relationship (Taurisano et al., 2023).

## Future directions

Caring for cancer survivors is a complex task with multiple psychological facets. In this study, we have proposed a 2 × 2 model of interpersonal emotion regulation by caregivers for cancer survivors. The model considers how caregivers may not only seek to make cancer survivors feel better but also, at times, may actively strive to make cancer survivors feel worse, even when caregivers have cancer survivors' best interests at heart. Moreover, caregivers may sometimes regulate cancer survivors' emotional states for reasons that are at least somewhat self-serving rather than purely altruistic. Interpersonal emotion regulation by caregivers can thus be supportive (survivor-centric hedonic), paternalistic (survivor-centric counter-hedonic), instrumental (caregiver-centric hedonic), or assertive (survivor-centric counter-hedonic).

Each of these four types of interpersonal emotion regulation entails trade-offs between specific psychological costs and benefits. For instance, the supportive type may allow cancer survivors to feel better but may also create undesirable emotional dependencies (Helgeson and Fritz, 2000). The paternalistic type may promote cancer survivors' long-term interests but may also lead cancer survivors to experience some amount of emotional discomfort (Seibel et al., 2023). The instrumental type may prevent immediate emotional distress in cancer survivors but often creates more psychological distance between cancer survivors and caregivers (Langer et al., 2009). In addition, the assertive type may prevent exhaustion among caregivers but tends to come at the expense of cancer survivors' immediate emotional needs (Chen et al., 2023). These trade-offs merit attention in the future research. Furthermore, it would be insightful to know whether and how caregivers can flexibly switch between or combine the four types of interpersonal emotion regulation. Such flexibility may be vital for the mental health and wellbeing of caregivers and cancer survivors (Kashdan and Rottenberg, 2010).

Our selective review of the literature found preliminary empirical support for the types of processes that are postulated by the 2 × 2 model of interpersonal emotion regulation by caregivers for cancer survivors. Nevertheless, it is important to keep in mind that the relevant empirical studies were not specifically designed to test the 2 × 2 model. The contribution of the present article is, therefore, primarily conceptual. Future research is needed to examine the 2 × 2 model across diverse caregiving contexts and cultural backgrounds to verify its applicability and robustness.

## Conclusion

Psychological care for cancer survivors is challenging. To meet this challenge, it is vital to consider not only the perspective of survivors but also that of caregivers. Addressing both perspectives may promote understanding between caregivers and cancer survivors, fostering the development of more mutually beneficial relationships.

## Author contributions

ZZ: Conceptualization, Funding acquisition, Resources, Writing – original draft. KH: Supervision, Validation, Writing – review & editing. SK: Funding acquisition, Supervision, Visualization, Writing – review & editing, Conceptualization.

## Footnotes

^1^Zeng, Z., Holtmaat, K., Jia, X., Burchell, G. L., Verdonck-de Leeuw, I. M., and Koole, S. L. (under review). *Interpersonal Emotion Regulation and Mental Health Among Cancer Survivors: A Systematic Review*.
